# Proceedings of the Michigan State Dental Society

**Published:** 1882-07

**Authors:** 


### PROCEEDINGS.

### PROCEEDINGS OF THE MICHIGAN STATE DENTAL SOCIETY.

The twenty-seventh annual meeting of the Michigan State Dental Society began its sessions at the Russell House, in the city of Detroit, on Wednesday, March 29, 1882, at 7:30 o'clock, P. M. President Metcalf in the Chair. The meeting was organized, and some miscellaneous business, such as filling committees, etc., was transacted, and after some free discussion upon the condition and status of the society, adjourned to meet on the following morning.

THURSDAY MORNING SESSION.

The meeting opened with many additional members in attendance. Dr. Shattuck, of the Committee of Censors, reported in favor of the election to membership of F. O. Gilbert, Bay City; C. S. Case, Jackson; W. H. Miller, Muskegon; A. W. Eldridge, Big Rapids; B. J. De Vries, Holland; Harriet L. Martindale, Grand Rapids; C. F. Porter, Bay City.

When Dr. Martindale was declared elected, Dr. Dorrance, who occupied the Chair, spoke of the new experience in the reception of a lady to membership, and, asking Miss Martindale to rise, introduced her to the members present. After some announcements, Dr. Shattuck moved to take up the discussion of some of the papers read at the previous meeting.

Dr. Whiting, speaking of six-year molars, said that if parents have good teeth their children will inherit them. Our great destroyer of teeth, at the present day, is the use of grape sugar with which all syrups and confectionary are adulterated. This is because sulphuric acid is used in its manufacture.

Dr. Clawson thinks that six-year molars require same treatment as other teeth, and that sulphuric acid does no more harm than other acids.

Dr. Shattuck said that light decay is caused by nitric acid and dark decay by sulphuric acid, and that six-year molars are usually affected by light decay.

Dr. Barrett believed that mankind had use for the entire set of teeth, and disapproved of the removal of any of them. The six-year molar is directly opposite the great salivary duct and plays an important part in mastication. The decay of teeth is nothing but chemical solution of the lime salts. The process of making starch into grape sugar is analogous to the change starch undergoes in the human system. There is nothing harmful in glucose, beyond that which comes from leaving fragments of it between the teeth, there to undergo fermentation, by which citric acid is generated. Candy, made from it, is healthier than that made from cane sugar.

Dr. Whiting objected to this on the ground that it is used only for the adulteration of cheap sugars and syrups and that not a pound of it could be found on sale under its own proper name.

Dr. Brophy insisted that it is lactic acid that destroys teeth, and especially the six-year molars, instead of acetic acid, and explained the chemical action which goes on in this matter collected between the teeth.

Dr. Talbot said that the impression had gone abroad that sulphuric acid enters largely into the manufacture of candies from glucose. If this were true the copper vessels in which candy is made would be speedily destroyed.

Dr. Perry said that while it was difficult many times to save the six-year molars he always tried to preserve them as long as possible but not at the sacrifice of the other teeth.

Dr. Douglass believed that the extraction of the six-year molars at ten years of age allowed the twelve-year molars to be better situated.

Dr. Waye believed in saving the first molar, if possible, as the creator, who was wiser than the dentist, evidently so designed it. Much depends on the care taken of these teeth during the first period of eruption, and parents should be urged to attend to this matter. When asked what filling he would use for deciduous teeth, answered, "That material which best answers the purpose."

Dr. Hunter said that what is often thought to be absorption of the roots of deciduous teeth is necrosis instead. Are we working for the benefit of our patients and the coming generation—are we students or mere mechanics? There are causes for the decay of these teeth and we should study them.

Dr. Owen said the jaw could not be well formed if the deciduous teeth were removed too early.

Dr. Taft said "six-year molar" was a misnomer. It should be called the first permanent molar so that there may be no doubt as to what is meant. The treatment which is proper for any other tooth is appropriate for this one. The extraction of any tooth is liable to change the position of neighboring teeth; possibly more noticeably so in the case of this molar than any other. When these teeth come into the care of the dentist, they are usually much decayed, and the first thing to be decided is whether or not they can be saved. It would be better to expand the jaw rather than extract the tooth for lack of space.

Dr. Douglass said that in the course of a long practice in the same place, he had had excellent opportunities for observing the development of the teeth, and could testify the success of filling roots of molars after the crowns had been lost.

The Chairman then appointed Drs. Douglass, Taft and Thomas a committee to prepare resolutions of respect to the memory of Dr. Hawxhurst. Adjournment until 2 P. M.

### AFTERNOON SESSION.

After some preliminary business, Dr. Taft re-opened the discussion of the morning by urging the members, and especially the younger ones, to pay particular attention to a proper nomenclature, his remarks being prompted by the frequent use, during the morning, of the term "six-year molar."

Dr. Dorrance said that the first permanent molars were as important as any other teeth. Their loss entailing a loss of grinding surface, a loss of support to the neighboring teeth and results in the shortening of the jaw. They should be saved if possible.

Dr. Taft said that twenty years ago he had filled these teeth with tin, thinking they would last but a short time, but many of them are in good condition to-day, having become better solidified and more perfect.

Dr. Barrett asked if the roots of deciduous teeth are absorbed when the pulp dies.

Dr. Brophy said that they were disintegrated by caries or necrosis, become liquified and then absorbed. When decay commences early in the deciduous incisors, he favors separation, using the file if necessary. Children should use the tooth brush as soon as they have teeth to brush, and should keep up the practice through life.

Dr. Harroun said that it was difficult to prescribe a uniform course of treatment for a diseased tooth. Judgment is required in all cases.

Dr. Wilson read a paper on "Treatment of Deciduous

Teeth." He spoke of the care to be given young children when the teeth were being erupted; the diseases from which they suffered at those times and the remedies.

Dr. Harlan did not believe that the absorption of the roots of deciduous teeth was due to the pressure from the crowns of the permanent teeth.

Dr. Taft said that at the proper time nature caused a formation of a little pulpy mass, flesh like, below the roots of the deciduous teeth. From this exudes a liquid that dissolves these roots and the rising of the crown of the permanent tooth slowly elevates this body or organ until the entire root is dissolved.

Dr. Barrett said that the English school inclines to the theory of necrosis, the German to dissolution and the French to a return to the original elements. He does not believe in the popular dissolving theory, but is of the opinion that after any tissue has performed its mission, it returns to its embryonic condition. He never found anything laid down in books that he did not have to modify in practice. The books say that at the time of dentition, children are subject to stomachic derangements This is true, but one is not the result of the other.

The growing of the teeth is a natural physiological development, and no more causes illness than the growing of hair or toenails. The stomachic troubles are caused by another natural change—that change in the digestive organs from the condition when the food is milk, and that when the child begins to consume other and coarser food.

The Board of Censors reported favorably for election to membership Drs. Damon, Rowly, Wilson, and Williams.

Dr. Barrett, of Buffalo, N. Y.; Drs. Harlan, Talbot, and Brophy, of Chicago, Ill., and Dr. and Mrs. Kate C. Moody, D. D.S., of Mendota, Ill., were elected to honorary membership. Adjourned until evening.

EVENING SESSION.

After the reading of letters of regret, and attending to other matters of business, the election of officers took place, with the following result:

President—A. T. Metcalf, Kalamazoo.

First Vice-President—W. H. Dorrance, Ann Arbor.

Second Vice-President—F. W. Clawson, Detroit.

Secretary– J. B. McGregor, Port Huron.

Treasurer—J. Lathrop, Detroit,

The consideration of topics for discussion being then in order, Dr. Taft began by saying that the teeth of children need exercise; need to be used in mastication, for unless this is done the teeth are soft and imperfect, and the jaws not properly expanded and strengthened. Tobacco chewers usually have strong jaws.

Dr. Whiting said that there was needed a course of dental gymnastics, or else the time is not far off when we will have no teeth, but will become a race of suckers.

Dr. Thomas, said that some years ago a committee had been appointed to confer with the publishers of school readers with a view of having them insert some short, terse articles upon the teeth, and also hygiene of the mouth. He would suggest that some further steps be taken in that direction.

FRIDAY MORNING SESSION.

After the usual preliminary business was attended to, Dr. McGregor read a paper on "The Relation of Dentistry to Medicine."

Drs. Harroun and Benedict, in speeches following this paper, deplored the great ignorance of dental matters as existing among physicians.

Dr. Barrett, of Buffalo, vigorously attacked the common idea that dentistry, as generally practiced, is a specialty of medicine. The only way into the medical profession was through the door of a medical college. Dentists should stand on their own merits, and not hang on the outskirts of another profession.

Dr. Watling said that Dr. Barrett was under a misapprehension as to the course of study in dental colleges, at least in the Dental Department of the University of Michigan. There the student studies anatomy as the medical students do, attends lectures on gynæcology, the practice of medicine, etc., and with a little additional study could become regular M.Ds.

Dr. Wilson pointed out the fact that a section of dentistry had been established in connection with the National Medical Association.

Dr. Barrett approved of the connection of dental and medical colleges, hoping that thus, might the separate institutions be driven to the wall.

The argument became quite spirited, some members claiming that every one who practiced the art of healing is a doctor, no matter what portion of the human frame he treats, and no matter what are his mental or scientific attainments, while others thought that the treatment of diseases of the teeth as separate and distinct from the practice of medicine as the treatment of diseases of the feet; that doctors of medicine know no more about diseases of the teeth and mouth than dentists know about diseases of the vital organs. Adjournment.

AFTERNOON SESSION.

"The Mounting of Artificial Crowns" occupied the attention of the members nearly all the afternoon, the different speakers explaining their various methods of performing this interesting operation.

Dr. Douglass, from a special committee, reported resolutions of regret, as follows:

*Whereas*—The information has come to us that Dr. D. C. Hawxhurst died in Paris, France, February 16, and,

*Whereas*—It seemed good to Him who doeth all things well, that our friend and professional brother should thus early be removed by death.

*Resolved*—That by his death we have lost a fellow laborer in dentistry, of rare attainments for one of his years, whose whole soul was devoted to his profession. Possessing, as he did, an amiable, courteous and generous disposition, he was known only to be loved by all. We shall fondly cherish his memory and greatly deplore his loss.

The resolutions were adopted.

At the evening session, President Metcalf read a carefully prepared and interesting history of dentistry in Michigan, covering a period of more than forty years. He said the first dentist to settle in the State was Dr. Douglass Houghton, in 1831. He settled in Detroit. Dr. J. H. Farnsworth had a practice here continuously for forty-five years. The first to settle in the interior of the State was Dr. Joseph Mansfield, of Niles, in 1837. From the Detroit City Directory of 1845 Dr. Metcalf copied Dr. Bailey's card. It read: "Dr. Bailey, from his former experience and uniform success, while soliciting a share of public patronage, has confidence in saying his operations shall be performed in the latest and most approved manner, and always with the least possible inconvenience to his patients; and by the use of Dr. Ware's nerve-destroyer, he is enabled to remove all sensibility in those teeth otherwise too sensitive to fill, and to destroy the nerve, when exposed, without pain or injury to the teeth. This, in fact, renders operations painless" I wish we could do that now, added the speaker.

Dr. Metcalf told of the organization of the Society in 1856, at which he was present. He was pleased to state that most of the charter members were present at this meeting. He traced the history of the founding of the Dental Department of the University of Michigan; the "vulcanized rubber suits," and the present contest with C. C. Burt, of Jackson, growing out of the same subject.

The paper was well appreciated.

With Dr. Clawson in the chair, Dr. Metcalf offered a series ot resolutions as a substitute for those offered by him at the last meeting of this Association in 1881, providing for the abolition of the chair of Mechanical Dentistry at University of Michigan. The following substitute was offered:

*Whereas*––That part of our practice known as mechanical dentistry, when so conducted as to give to patients the highest attainable results, has become so intricate and complicated that it can not be properly learned in the time now devoted to the teaching of it in our dental colleges, and

*Whereas*—In consequence of the insufficient instructions now given in it tends to cheapen, belittle and degrade it; therefore.

*Resolved*—That the officers of the Association and the visitors to the Dental Department of the University of Michigan are hereby instructed to make all proper efforts to have mechanical dentistry taught as a distinct calling, and when students have become proficient in its various departments that they be entitled to receive a certificate of qualification to practice it, regardless of their qualification to practice dental surgery.

The resolution was laid on the table, and the Association then adjourned."

THE BANQUET AND SPEECHES.

Shortly after 9 o'clock the members of the Association, and invited guests sat down to a banquet specially provided for the occasion by the proprietors of the Russell House, and one that was a credit to even these favorite caterers to the public. The supper over, toasts became the order of the occasion, and were as follows:

The City of Detroit—May her beauty ever be equaled by her prosperity. Response by Dr. Cahoon.

The Press. Response by F. J. Irland.

The Pioneers of Dentistry. Smile at their first small ventures as we will, the schoolboy's copy shapes the scholar's hand, and their grateful memory fills our hearts to-day. Response by Dr. C. B. Porter.

The relation that medicine bears to dentistry. "All are but parts of one stupendous whole." Response by Dr. W.W. Allport, of Chicago.

Dental literature. Diffusiveness without concentration should be avoided. Words may be fluently used without conveying ideas. Responses by Dr. Eugene S. Talbot, of Chicago, and Dr. Barrett, of Buffalo.

Dental Education. A little learning is a dangerous thing. "Drink deep, or taste not the Pyerian spring." Response by Dr. J. Lathrop.

Dental hygiene in our public schools. Response by Prof. Hailmann, of the Board of Education.

The past, present, and future of the dental profession. Response by Dr. J. A. Robinson.

Dental legislation. "The mills of the gods grind slowly." Response by Prof. Taft, of the University.

Dental societies. "In union there is strength." Response by Dr. C. H. Haroun, of Toledo.

The relation and influence of the older practitioners on the younger." Response by Dr. T. W. Brophy, of Chicago.

Dental students. "In the long run fame finds deserving men. The lucky wight may prosper for a day, but in good time true merit leads the van." Response by Dr. W. H. Dorrance, of Ann Arbor.

"Mechanical dentistry, whose end both at first, and now, is to hold the mirror up to nature." Response by Dr. E. C. Moore.

Our duties to our patients. "Let patience have her perfect work." "Words fitly spoken are like apples of gold." Response by Dr. J. Hamilton Thurston, of Jamestown, N. Y.

Dental colleges. "May their advance be steady, unhindered by retreat." Response by Dr. J. A. Watling, of Ann Arbor.

The dentist's leisure hours. "Man never is, but always to be blessed." Response by Dr. H. Cowie.

